# Effect of self-rated health status on functioning difficulties among older adults in Ghana: Coarsened exact matching method of analysis of the World Health Organization’s study on global AGEing and adult health, Wave 2

**DOI:** 10.1371/journal.pone.0224327

**Published:** 2019-11-05

**Authors:** John Tetteh, Robert Kogi, Anita Ohenewa Yawson, George Mensah, Richard Biritwum, Alfred Edwin Yawson

**Affiliations:** 1 Department of Community Health, School of Public Health, College of Health Sciences, University of Ghana, Accra, Ghana; 2 National Cardiothoracic Centre, Korle-Bu Teaching Hospital, Accra, Ghana; 3 Department of Epidemiology and Biostatistics, School of Public Health, University of Health and Allied Sciences, Hohoe, Ghana; 4 Department of Anesthesia, Korle Bu Teaching Hospital, Accra, Ghana; 5 Department of Biostatistics, School of Public Health, College of Health Sciences, University of Ghana, Accra, Ghana; University of Indianapolis, UNITED STATES

## Abstract

**Background:**

Functional difficulty assessment has been proven as a key factor in the health evaluation of adults. Previous studies have shown a reduction in health and functional difficulties with increasing age. This analysis was conducted to quantify the effect of poor self-rated health on functional difficulty among older adults in Ghana.

**Method:**

This analysis was based on the World Health Organization Study on Global AGEing and Adult Health in Ghana for older adults 50 years and above. Fifteen standard functioning difficulty tools were extracted and used for the analysis. Three predictive models with the Coarsened Exact Matching method involving Negative Binomial, Logistics and Ordered logistic regression were performed using Stata 14.

**Results:**

Overall, the prevalence of poor Self-rated health was 34.9% and that of functional difficulties among older adults in Ghana was 69.4%. Female sex, increasing age, being separated, having no religious affiliation, not currently working and being underweight were associated with and significantly influence poor Self-rated health [AOR(95%CI)p-value = 1.41(1.08–1.83)0.011, 3.85(2.62–5.64)0.000, 1.45(1.08–1.94)0.013, 2.62(1.68–4.07)0.000, 2.4(1.85–3.12)0.000 and 1.39(1.06–1.81)0.017 respectively]. In addition, poor Self-rated health and geographical location (rural vs. urban)significantly influence functioning difficulties among older adults in Ghana as predicted by the three models [Negative Binomial: PR(95%CI) = 1.62(1.43–1.82), Binary logistic: AOR(95%CI) = 3.67(2.79–4.81) and ordered logistic: AOR(95%CI) = 2.53(1.14–2.03)].

**Conclusion:**

Poor SRH is more pronounced among older adult females in Ghana. Some determinants of poor SRH include; age, geographical location (urban vs. rural), marital status, religion, and employment status. This provides pointers to important socio-demographic determinants with implications on the social function of older adults in line with the theme of the national aging policy of 2010, ‘ageing with security and dignity’ and ultimately in the national quest to achieve the Sustainable Development Goals by 2030.

## Introduction

Self-rated health (SRH) has been shown to be a reliable predictor and a measure of health outcomes including mortality, functional difficulties, and chronic diseases. It is also specifically influenced by the somatic experience that generates specific health conditions [1–5]. Self-rated health is a generally accepted health status rating assessment that captures health rated information of the individual and is mostly used in adult surveys to assess the health status of adult populations [4]. Individual SRH is a subjective well-being which measures health status and is not merely the absence of disease or infirmity but also captures the main component of the definition of health by World Health Organization (WHO) including the physical, mental and social well-being of the individual [6].

Previous studies have indicated a strong correlation between SRH and a wider context of health outcomes including functional difficulties [7–9]. Older adults are most vulnerable due to developmental and physiological processes, which are major causes of reduction in their quality of life [10, 11] with biological, social, and psychological dimensions [10]. Globally, functional difficulties may result from acute or chronic diseases, injuries, mental or emotional challenges, and alcohol or drug use [12]. Moreover, physical functional difficulty measures are not only associated with clinical and subclinical age-related changes but are also able to predict future health-related events [13, 14].

Older adults with functional difficulties in either mobility or basic activities have higher odds of reporting poor SRH [15]. Data exist to indicate that SRH is influenced by demographic variables (such as sex, age, and working status); social factors (such as social networks and family functioning); biological factors (including the presence of illnesses and use of medications); mental factors (including anxiety, depression, dementia or grief); and functional determinants (such as physical and basic daily activities) [16].

Demographic factors including increasing age are associated with a reduction in health and functional difficulties and increase the older persons’ demand for health care and other social services[17]. Older adults with little or no education have poor SRH strongly correlated with functional difficulties [4].

In the developing world, previous studies have indicated that older adults who had never worked in a lifetime, geographical location (rural vs. urban) and those with functional difficulties are more likely to report poor health [1].

### Self-rated health in developing countries

In sub-Saharan Africa, it has been established that age and sex play fundamental roles in SRH among urban dwellers whereas economic well-being was identified to be associated with SRH among rural dwellers [18]. Among older adult women in Senegal, hypertension, community membership and religion have been shown to be associated with SRH [19]. Interestingly, Marcia and colleagues in 2012 reported that, SRH was significantly correlated with felt age but not with ideal age and that, the more Senegalese older adults rated their health positively, the younger their felt age. However, Senegalese who rated their health as below average, belonged to the old age group as opposed to self-rated better health [20].

In South Africa, males report relatively good health compared to females and that, trust was also identified to be positively associated with self-reported good health. However, neighborhood social capital, personalized trust, and individual community service group membership were negatively associated with reporting good health in some parts of South Africa [21, 22].

Similarly, close to 30.0% of older adults in Nigeria self-rated their health as poor and that, being married, engagement in work, absence of morbid conditions and higher levels of education were significant predictors of good SRH in older adults in Nigeria [23]. In addition, in Nigeria, a reported 8.4% of postmenopausal women self-rated their health as poor/fair, and among these, involvement in moderate to vigorous physical activity (PA) was positively related to SRH [24]. Another study in Nigeria observed that SRH was negatively associated with physical impairments [25].

In another West African country, Burkina Faso, Onadja and colleagues in 2013 established that, poor SRH was strongly correlated with chronic diseases and functional difficulties and that functional difficulty on poor SRH increased with age [26]. The overall prevalence of poor SRH was shown to be 38.5% in Burkina Faso [27].

In Ghana, the index country for the current analysis, Depbuur and colleagues in 2015, observed in one of the districts (Kassena-Nankana) younger adults rated their health status relatively better compared to the older adults. In that study, it was demonstrated that functional ability and sex were significantly associated with SRH status i.e. adults with higher levels of functional limitations were more likely to rate their health as poor [28]. Two years later across the whole country, Fonta et al (2017) reported in Ghana that about 20.1% of older adults rated their health status as poor, and self-rated poor health was relatively higher among older adults. In addition, older adults with one or more chronic health conditions were at higher risk of reporting poor health. In 2018, Gyasi and Philips examined the association between SRH and functional decline in older Ghanaian adults and found that sex was a key factor i.e. females reported more functional decline compared to males [29].

Overall, evidence exists that there is a relationship between demographic characteristics and functional difficulties, which are direct predictors of poor SRH among older adults [3, 5, 13–15, 17]. Sex differential, place of residence and other demographic variables as a predictor of SRH have been established [18–33]. What is not clearly articulated is a direct comparison that quantifies how much effect poor SRH has on functional difficulty. In Ghana, there is limited data to assess SRH and how it influences functional difficulty among older adults. It is imperative to examine how Ghanaian older adults (50 years and above) rate their health and how it influences their abilities. This analysis was thus conducted to quantify the effect of SRH on functional difficulty among older adults in Ghana.

## Methods

### Study setting

The WHO Study on Global Ageing and Adult Health (SAGE) wave 2 for Ghana was conducted in 2014–2015, as part of the multi-country study on aging.

### Study participant

The dataset of the WHO Study on Global Ageing and Adult Health (SAGE) wave 2 for Ghana was used for this analysis. SAGE is longitudinal data on the health and well-being of adult populations, and the aging process, through primary data collection and secondary data analysis. SAGE Wave 2 was from 2014 to 2015 in six lower-to-middle income countries including; China, Ghana, India, Mexico, Russian Federation, and South Africa [34]. Two target populations were used in SAGE Wave 2 which include a large sample of persons aged 50 years and older (focus group for SAGE) and a smaller comparative sample of persons in the age group (aged 18–49 years). Households were classified into mutually exclusive categories where one or more persons aged 50 years and older were selected from households classified as “50+ households” and one person aged 18–49 years from a household classified as an “18–49 household”. In the older households, all persons aged 50 years and older were invited to participate whiles proxy respondents were identified for respondents who were unable to respond for themselves. Multistage cluster sampling design was used for Ghana wave 2 with 250 Primary Sample Unit and 20 strata [34, 35]. Detailed study design and procedure for data collection adopted for the SAGE survey is in Kowal et al. (2012) [36].

In all, 4735 respondents were involved in the SAGE wave 2 with the inclusion of both adults and those in 18–49 years. Based on the objective of the study, those below the age of 50 years, missing or not applicable responses, and those designated as don’t know responses were excluded. A total sample size of 3339 older adults, ≥50 years was used for the analysis.

### Dependent variables

There are two dependents variables that were taken into consideration; self-rated health (SRH) and functional difficulties. In self-rated health, respondents were asked to rate their health at the time of data collection. The question used was “In general, how would you rate your health today?” Respondents rated their health from 1 “Very good”, 2 “Good”, 3 “Moderate”, 4 “Poor”, 5 “Very poor”. For the purpose of analysis, response 1 and 2 were merged and re-categorized as 0 “Good SRH” and response 3–5 were also merged and re-categorized as 1 “Poor SRH”. This approach of re-categorization of SRH status was adopted to conform to the study objective and the approach adopted in different studies [18, 31, 37]. As indicated in literature, SRH is considered as a subjective indicator and psychological tool which captures not only overall current health status but also historical, current, and future hospital records [29, 32]. SRH specifically covers the physical, emotional and personal components of health [29] which has now gained attention worldwide. SRH has been established in literature as a good predictor of future health status [30].

Functional difficulties (FD), was assessed in SAGE Wave 2 with the question, “In the last 30 days, how much difficulty did you have in …” This composite question included 15 standard sub Likert-scale questions relating to standing for long period, household responsibilities, joining community activities, concentration on doing something, walking for a long distance, washing the whole body, getting dressed, day to day work, carrying things, eating, getting up from lying, getting to and using the toilet, getting where you want to go, going out of home and emotional effect by health condition. For internal consistency and reliability, Jann Stata module to compute Cronbach's alpha for weighted data was used due to the design of the SAGE study [38]. The overall test of reliability for functioning difficulty domains is very high and of good quality to measure FD (α = 0.93). The items all tapped into the same concept (see Table 1).

**Table 1:** Functioning difficulty reliability test for fifteen items.

Item	Total observation	Sign	Item-test correlation	Item-rest correlation	Average inter-item covariance	alpha
Standing for long period	3326	+	0.76	0.71	0.09	0.92
Household responsibilities	3312	+	0.76	0.72	0.09	0.92
Joining community activities	3295	+	0.74	0.68	0.09	0.93
Concentration on doing something	3331	+	0.75	0.70	0.09	0.92
Walking for a long distance	3269	+	0.79	0.74	0.09	0.92
Washing whole body	3324	+	0.65	0.61	0.09	0.93
Getting dressed	3321	+	0.61	0.56	0.09	0.93
Day to day work	3294	+	0.77	0.72	0.09	0.92
Carrying things	3181	+	0.77	0.72	0.09	0.92
Eating	3320	+	0.53	0.48	0.10	0.93
Getting up from lying	3322	+	0.64	0.58	0.09	0.93
Getting to and using the toilet	3320	+	0.66	0.61	0.09	0.93
Getting where you want to go	3316	+	0.75	0.70	0.09	0.92
Going out of home	3316	+	0.71	0.66	0.09	0.93
Emotional effect by health condition	3316	+	0.77	0.72	0.09	0.92
**Test scale**	** **	** **	** **	** **	**0.09**	**0.93**

These Likert-scale questions have the same graded responses as 1 “None”, 2 “Mild”, 3 “Moderate”, 4 “Severe”, 5 “Extreme” and 9 “Not applicable” (designated as missing). Descriptive analysis was run on all the 15 questions. The overall functional difficulty was reclassified; response 1 was replaced as “None = 0” and response 2–5 were replaced as “Yes = 1” for all questions.

An index variable was generated for the 15 questions and a score was graded ranging from 0–15 with a mean (standard deviation) score of 4.44(4.61). Raw scores were analyzed as continuous variables and graded (recoded into four categories as 0 “, 1–7, 8–14 and 15) scores and analyzed as categorical variables for the inferential statistical analysis.

### Independent variable

#### Demographic variable

The sex variable was coded as “male and female”, age was categorized as “50–59, 60–69, 70–79 and 80 and above”. Marital status was categorized as “Never married, married, separated and widowed” whiles religion was classified as None, Christian, Islam and primal indigenous (which includes Buddhism, Chinese traditional religion, Hinduism and others). Place of residence was indicated as “Urban” and “Rural” whiles work status was also categorized into “Yes and No”. Body Mass Index (BMI) was classified as “Underweight Normal, Overweight and Obesity”. These demographic variables were considered in line with observations by Lim et al (2007) that, some of these demographic variables are associated with poor SRH [37].

### Data analysis

Preventing bias estimations was a key factor considered during the analysis of the complex SAGE survey data to reduce bias and improved our estimates. The complex nature of the study design is related to the Primary Sampling Units, Stratification, and Individual Sampling weights. Descriptive statistics involved two-way observational weighted row percent table involving independent variables associated with SRH and analyzed with a corrected chi-square. In addition, inferential statistics involving logistics regression (weighted estimation) was conducted.

Coarsened Exact Matching (CEM) method of analysis was conducted to improve and reduce imbalances in the estimation of an effect between treated (adults with poor self-rated health) and control groups (adults with good self-rated health). In order to control for some or all of the potentially confounding influence of pretreatment control variables, the CEM method (a Monotonic Imbalance Bounding (MIB) matching method between the treated and control groups) was applied. [39]. In order to improve and reduce imbalances in estimating the effect of poor SRH on FD, identified association and predictor variables with poor SRH (including; sex, age, marital status, religion, working status, region, and BMI) were modeled as a weighted variable using CEM.

Table 2 shows that overall, 64% of imbalance existed among the predictors of poor SRH before matching. However, after CEM matching, imbalances reduced to almost 0% (1.883E-15). After preprocessing the data with CEM, a sensitivity analysis was applied involving Negative Binomial, Logistics and Ordered logistics regression controlling for weighted with CEM estimations to estimate the effect on poor SRH on functional difficulty were conducted. The choice of the analytical procedure was based on the principle of coarsening the predictor’s variables, exact match on the coarsened data which reduces imbalances between and within predictors of poor SRH, and finally performing analysis on the matched data to estimate the robust standard error. CEM reduces covariate imbalance for the subsequent determination of a treatment effect which is poor SRH. The CEM another form of propensity score matching, allowed us to specify matching levels within samples of each poor SRH predictor variable. This ensures the degree of balance in the matching variables at the lowest level [40]. CEM is preferable to other matching procedures in terms of producing a balanced sample and reducing model dependence and estimation error applied in contemporary health, social and epidemiological research [40–42].

**Table 2:** CEM weighted balance report before and after matching.

Matching variable	Before matching		After matching	
	L1	mean	L1	mean
Sex	0.07	0.07	2.50E-16	-2.90E-15
Age	0.26	0.62	6.20E-16	4.40E-15
Marital status	0.18	0.54	4.60E-16	9.30E-15
Religion	0.04	-0.13	4.60E-16	5.30E-15
Working status	0.26	0.26	4.20E-16	1.30E-15
Region	0.13	-0.48	1.40E-15	2.60E-14
BMI	0.07	-0.11	6.70E-16	-1.80E-15
**Overall**	**0.64**		**1.88E-15**	

NOTE: Total match sample among the treatment (POOR SRH) = 677. Total match sample among controls (Healthy) = 1153. E = exponent

Upon preprocessing the data with CEM, a sensitivity analysis was applied involving Negative Binomial (NB), Logistics and Ordinal logistics regression using raw and reclassification scores respectively for estimating the probability of functional difficulty by applying CEM weights with robust standard estimations among poor SRH and adjusting for geographical location (rural vs. urban) among older adults in Ghana. Negative Binomial regression was applied based on the assumption that functional difficulty was assessed on raw counts which were positive integers with over-dispersion, that is, the variance exceeds the mean (σ^2^ vs μ = 21.2 vs 4.4). Binary logistic regression was applied because the outcome variables were dichotomized (for SRH 1 “poor SRH” and 0 “Healthy” whiles for FD 1 “Yes” and 0 “None”). In addition, ordered logistic regions was used due to the ordinal categorization of functional difficulty. The mean comparison test of all FD variables was also assessed among good and poor SRH by using t-test statistic from Linear combinations of parameters after mean estimation in Stata. A significant level was set at p-value<0.05. All analysis was carried out using Stata 14.

### Ethical requirements

This research used data from the WHO SAGE Ghana survey. SAGE was approved by the World Health Organization's Ethical Review Board (reference number RPC149) and the Ethical and Protocol Review Committee, College of Health Sciences, University of Ghana, Accra, Ghana. Written informed consent was obtained from all study participants.

## Result

This analysis was conducted among 3339 adults aged 50 years and above. Off this, a total of 1256 (34.9%) were found to be unhealthy per the inclusion criteria.

Overall, there is a prevalence of 34.9% of poor SRH among older adults in Ghana. As in Table 2, a sex differential in poor SRH exists; more in females (39.7%) compared to males (29.5%). Older ages and being widowed showed a relatively higher prevalence of poor SRH in the older adults (66.2% in those ≥ 80 years and 49.8% among the widowed (see Table 3).

**Table 3:** Demographic characteristics and prevalence of Self-rated health status among older adults in Ghana, SAGE Wave 2, 2014–2015.

Demographic variable	Total	Poor SRH	Design-based χ^2^
	N	n(%)	
Total	N = 3339	1256(34.9)	
Sex			23.50***
Male	1392(100)	470(29.5)	
Female	1947(100)	786(39.7)	
Age group			59.72***
50–59	12399100)	285(23.8)	
60–69	1060(100)	376(36.2)	
70–79	704(100)	376(54.2)	
80+	336(100)	219(66.2)	
Marital status			32.23***
Never married	109(100)	25(20.9)	
Married	1904(100)	591(29.0)	
Separate	396(100)	173(42.3)	
Widowed	930(100)	467(49.8)	
Religion			2.99*
None	110(100)	68(53.0)	
Christian	2398(100)	889(34.0)	
Islam	622(100)	217(33.8)	
Primal Indigenous	209(100)	82(38.5)	
Place of residence			1.46
Urban	1287(100)	469(33.1)	
Rural	2052(100)	787(36.5)	
Currently working			
Yes	2228(100)	638(26.6)	
No	1061(100)	601(54.9)	
Region			2.83**
Ashanti	540(100)	256(41.3)	
Brong Ahafo	360(100)	137(31.7)	
Central	432(100)	130(24.5)	
Eastern	260(100)	126(45.6)	
Greater Accra	304(100)	129(36.8)	
Northern	347(100)	133(35.1)	
Upper East	186(100)	55(30.5)	
Upper West	167(100)	33(23.4)	
Volta	311(100)	114(32.1)	
Western	432(100)	143(27.8)	
BMI			4.12**
Underweight	424(100)	214(45.9)	
Normal	1841(100)	665(32.4)	
Overweight	675(100)	232(34.2)	
Obesity	399(100)	145(37.7)	

* = p-value ≤0.05

** = p-value ≤0.01 and

*** = p-value ≤0.001

In addition, poor SRH was relatively higher among those with no religious affiliation (53.0%) and older adults residing in rural locations (36.5%). Older adults not working, experienced relatively higher poor SRH (54.9%). Table 3 demonstrates that older adults who were underweight (based on BMI estimates) had a relatively higher prevalence of poor SRH (45.9%) in Ghana. The observed differences in poor SRH among older adults in Ghana were significantly associated with sex, increasing age, marital status, religious affiliation, work status, and BMI (see Table 3).

Table 4 indicates that females have 1.41 chance of reporting poor SRH as compared to males [AOR(95%CI)p-value = 1.41(1.08–1.83)0.011]. Moreover, increasing age showed an increased odds for adults who were 80 years and over, 70–79 years and 60–69 years to have 3.85, 2.89 and 1.6 chance respectively of reporting poor SRH compared with those in the 50–59 years group [AOR(95%CI)p-value = 3.85(2.62–5.64)0.000, 2.89(2.17–3.86)0.000, and 1.6(1.18–2.16)0.002 respectively].

**Table 4:** Demographic predictors of poor SRH status among older adults in Ghana, SAGE Wave 2, 2014–2015.

Characteristics	Predictive factor	AOR	95% Confidence Interval	P-value
Poor SRH	Sex			
	Male	Ref		
	Female	1.41	1.08–1.83	0.011
	Age group			
	50–59	Ref		
	60–69	1.6	1.18–2.16	0.002
	70–79	2.89	2.17–3.86	<0.001
	80+	3.85	2.62–5.64	<0.001
	Marital status			
	Married	Ref		
	Never married	0.46	0.26–0.83	0.010
	Separate	1.45	1.08–1.94	0.013
	Widowed	1.31	1.01–1.69	0.044
	Religion			
	Christian	Ref		
	None	2.62	1.68–4.07	<0.001
	Islam	1.17	0.81–1.69	0.405
	Primal Indigenous	1.68	1.05–2.69	0.030
	Currently working			
	Yes	Ref		
	No	2.4	1.85–3.12	<0.001
	BMI			
	Normal	Ref		
	Underweight	1.39	1.06–1.81	0.017
	Overweight	1.11	0.81–1.52	0.504
	Obesity	1.07	0.71–1.60	0.762

Older adults without partners (separated, and widowed) were more likely to report poor SRH compared to those with partners (i.e. those who were separated and widowed were 1.45 and 1.31 times respectively likely to have reported poor SRH [AOR(95%CI)p-value = 1.45(1.08–1.94)0.013 and 1.31(1.01–1.69)0.044 respectively]

Interestingly, older adults with no religious affiliation and those in the primal indigenous religions had higher odds of reporting poor SRH compared with their Christian counterparts (i.e. those with no religion were 2.62 times and those with primal indigenous were 1.68 times likely to have reported poor SRH [AOR(95%CI)p-value = 2.62(1.68–4.07)0.000 and 1.68(1.05–2.69)0.030 respectively]. The work status of the older adult significantly influenced poor SRH. Older adults not working were 2.4 times likely to be associated with poor SRH [AOR(95%CI) = 2.4(1.85–3.12)0.000], compared to those who currently work (see Table 4).

In all, older adults classified as underweight were 1.39 times more likely to have poor SRH [AOR(95%CI) = 1.39(1.06–1.81)0.017].

Individual functional difficulty analysis indicates that as many as 53.5% of the older adults had some difficulty standing for a long period of time (in Table 5). In addition, 33.5%, of the older adults had difficulty in performing their household responsibilities. In all, very few of the older adults had severe and extreme difficulty in participating (joining) in community activities (3.5% and 3.3% respectively). However, over a quarter (24.2%) of the older adults had mild challenges, while 10.4% had moderate challenges in concentrating on doing something. In all, 45.2% of the older adults reported difficulty in walking for a long distance.

**Table 5:** Descriptive assessment of functioning difficulties among older adults in Ghana, SAGE Wave 2, 2014–2015.

In the last 30 days, how much difficulty did you have in …	Level of measurement					Total
	None	Mild	Moderate	Severe	Extreme	
Standing for long period	1349(46.5)	591(16.7)	897(23.7)	329(9.3)	160(3.9)	3326
Household responsibilities	2076(66.5)	671(18.4)	437(11.9)	79(2.1)	49(1.2)	3312
Joining community activities	1965(63.0)	602(17.9)	465(12.3)	131(3.5)	132(3.3)	3295
Concentration on doing something	1957(63.1)	875(24.2)	417(10.4)	63(1.8)	19(0.5)	3331
Walking for a long distance	1684(54.8)	395(13.2)	705(18.6)	320(9.2)	165(4.1)	3269
Washing whole body	2880(87.3)	328(8.9)	93(2.9)	19(0.8)	4(0.1)	3324
Getting dressed	2928(88.2)	295(9.0)	78(2.3)	13(0.3)	7(0.2)	3321
Day to day work	1905(61.0)	688(20.4)	509(13.2)	91(2.8)	101(2.5)	3294
Carrying things	1460(52.3)	432(13.0)	712(19.6)	299(7.6)	278(7.4)	3181
Eating	3071(92.6)	170(5.0)	58(1.7)	12(0.5)	9(0.2)	3320
Getting up from lying	2549(77.5)	557(16.4)	145(3.8)	56(1.9)	15(0.4)	3322
Getting to and using the toilet	2694(82.6)	466(13.0)	109(3.0)	43(1.3)	8(0.2)	3320
Getting where you want to go	2426(76.5)	619(16.5)	164(4.2)	66(1.9)	41(0.9)	3316
Going out of home	2575(80.3)	525(14.0)	133(3.5)	50(1.3)	33(0.9)	3316
Emotional effect by health condition	1704(57.6)	1013(26.7)	466(12.2)	106(2.8)	27(0.7)	3316

NOTE: Weighted results

As many as (87.3% and 88.2%) of the adults reported they do not have difficulty in washing their bodies and getting dressed respectively. Interestingly, a little over half (61.0%) of the older adults had no difficulties doing their day to day work, and 2.5% had extreme difficulties. In addition, 19.6% had moderate difficulty in carrying loads over the past 30 days.

**Fig 1:**
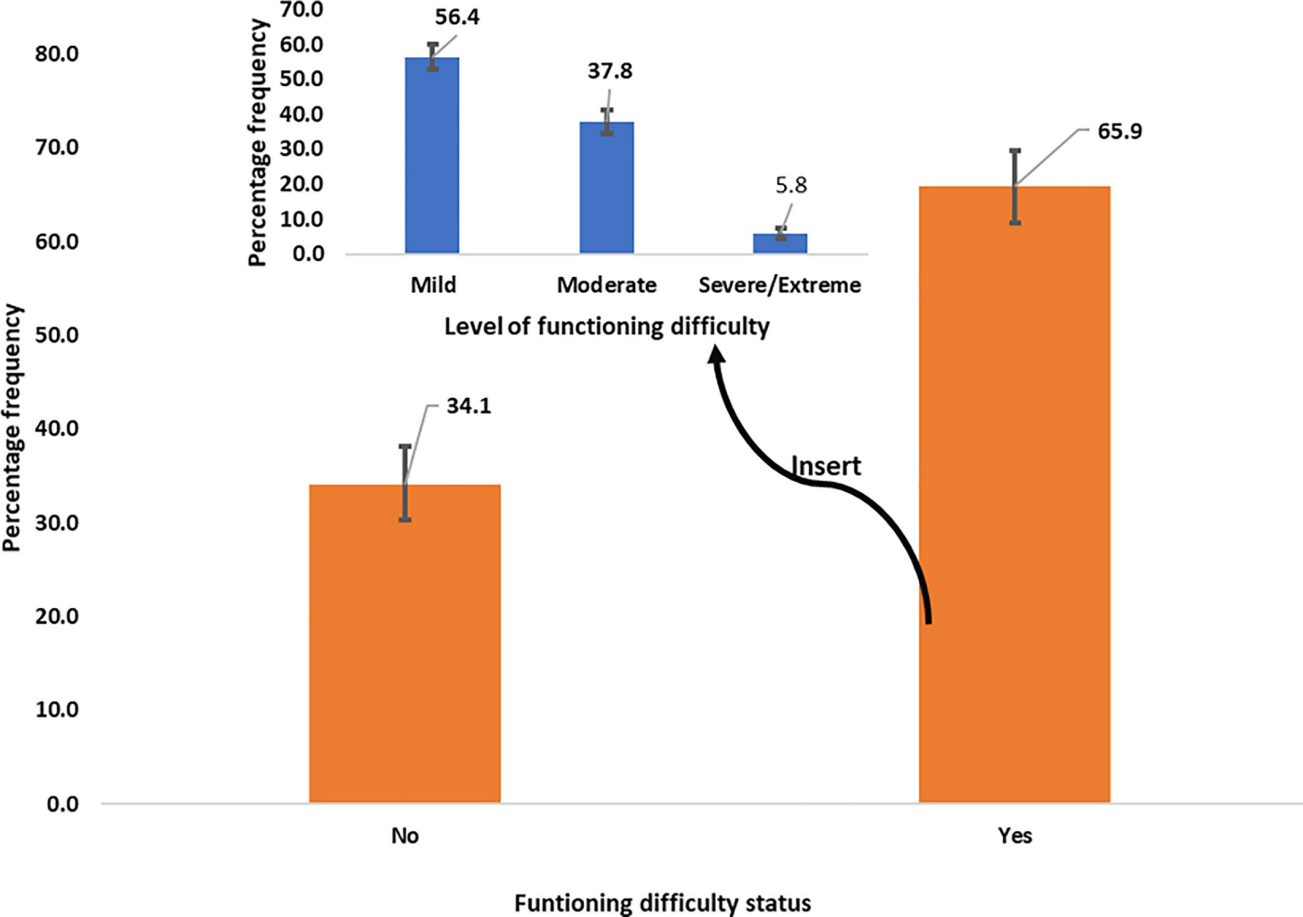
Status of functional difficulty among older adults in Ghana, SAGE Wave 2 from 2014–2015 showing 95% confidence interval. Insert showing levels of functional difficulties.

Overall, 65.9% of functional difficulties existed among older adults in Ghana and over half (56.4%) suffered mild functioning difficulties with a statistically significant differences in the proportions with mild, moderate and severe/extreme functioning difficulties, as in Fig 1.

Mean comparison shows that, poor SRH experienced a higher level of FD as compared to good SRH across the 15 structured FD questions (see Table 6). The mean difference predicts negative values and is statistically significant (p-value<0.001) as predicted by t-test statistic (see Table 6), implying that FD is mostly experienced among poor SRH older adults compared with good SRH.

**Table 6:** Mean comparison of functional difficulty among older adults in Ghana.

Functioning difficulty	Good SRH	Poor SRH	difference	t-test
Standing for long period	1.75	2.68	-0.93	-15.53***
Household responsibilities	1.29	1.98	-0.69	-16.25***
Joining community activities	1.39	2.18	-0.79	-14.13***
Concentration on doing something	1.33	1.88	-0.55	-13.32***
Walking for a long distance	1.7	2.41	-0.71	-9.94***
Washing whole body	1.09	1.32	-0.23	-8.05***
Getting dressed	1.08	1.3	-0.22	-9.11***
Day to day work	1.44	2.06	-0.62	-12.26***
Carrying things	1.73	2.66	-0.93	-12.49***
Eating	1.05	1.23	-0.18	-7.15***
Getting up from lying	1.18	1.56	-0.38	-10.01***
Getting to and using the toilet	1.16	1.38	-0.22	-7.73***
Getting where you want to go	1.21	1.59	-0.38	-9.37***
Going out of home	1.17	1.49	-0.32	-9.93***
Emotional effect by health condition	1.42	1.98	-0.56	-12.79***

NOTE

*** = p-value<0.001

Table 7 indicated below shows a significant pairwise correlation, where a significant positive relationship exists between SRH, place of residence, FD raw count, FD Binary category and FD Ordinal category among older adults (see Table 7).

**Table 7:** Significant pairwise correlations between SRH, place of residence, FD raw count, FD Binary category and FD Ordinal category with CEM weights.

	SRH	Place of residence	FD raw count	FD Binary category	FD Ordinal category
SRH	1				
					
	1830				
Place of residence	0.0733	1			
	0.002				
	1830	1830			
FD raw count	0.2438	0.063	1		
	<0.001	0.007			
	1830	1830	1830		
FD Binary category	0.2608	0.1103	0.6694	1	
	<0.001	<0.001	<0.001		
	1830	1830	1830	1830	
FD Ordinal category	0.249	0.0754	0.9243	0.8106	1
	<0.001	<0.001	<0.001	<0.001	
	1830	1830	1830	1830	1830

Controlling for sex, age, marital status, religion, work status, region, and BMI, there is a statistically significant association between poor SRH and place of residence and functioning difficulties (as illustrated in Table 7). Negative Binomial estimation predicts that poor SRH was 1.62 times likely to have functional difficulty [PR(95%CI) = 1.61(1.42–1.82)] compared to healthy older adults. Model 2 (binary outcome) predicts that, adults with poor SRH were 3.67 times likely to have reported functioning difficulty as compared to adults without poor SRH (logistic with CEM) [AOR(95%CI) = 3.67(2.79–4.81)] and the Ordered classification (Ordered logistic with CEM) also predicted that older adults with poor SRH were 2.52 times likely to be at the highest level of FD compared to low and middle level of FD [AOR (95%CI) = 2.52(2.03–3.12)]. There was a significant association between place of residence and functioning difficulty, such that, rural-dwelling older adults were 53% more likely to have reported FD (Logistic with CEM) [AOR(95%CI) = 1.53(1.14–2.03)]. Similarly, the rural-dwelling older adults were 29% more likely to report FD [AOR(95%CI) = 1.29(1.02–1.64)], as presented in Table 8.

**Table 8:** Sensitivity analysis showing Negative Binomial, logistic and ordered logistics regression with CEM at a 95% Confidence Interval of the effect of SRH on functioning difficulty among older adults in Ghana, SAGE Wave 2, 2014–2015.

Demographic variable	Sensitivity analysis		
	Raw count: Model 1	Binary outcome: Model 2	Ordinal outcome: Model 3
	NB with CEM	Logistic with CEM	Ordered logistic with CEM
	PR[95%CI]	AOR[95%CI]	AOR[95%CI]
**Health status**			
Good SRH	Ref	Ref	Ref
Poor SRH	1.62[1.43–1.82]***	3.67[2.79–4.81]***	2.52[2.03–3.12]***
**Place of residence**			
Urban	Ref	Ref	Ref
Rural	1.10[0.9–1.25]	1.53[1.14–2.03]**	1.29[1.02–1.64]*

NOTE: PR: prevalence ratio from multiple Negative Binomial regression model, AOR: adjusted odds ratio from binary logistic and ordinal logistic regression.

* = p-value ≤0.05

** = p-value ≤0.01 and

*** = p-value ≤0.001

## Discussion

Self-reported health (SRH) has proven to be a good indicator of objective health as well as a sensitive and robust predictor of health-related behaviors and health care demand [43]. This analysis found the prevalence of poor SRH among older adults in Ghana to be 34.9%. This value is relatively higher than that reported by Campinas, where 10.9% of negative self-rated health was observed among older adults [44]. However, a study in Mozambique reported a relatively higher prevalence of 54% in respondents aged 40 years or more [45].

Previous studies have indicated that females tended to self-report their health more negatively compared to their male counterparts [46]. This observation is congruent with the findings from this analysis which showed that older adult females are 1.41 times more likely to report poor SRH compared to males. It is also in agreement with what was reported in the European Studies on Aging (CLESA), (a longitudinal cross-national comparison study) which observed that in all countries, except Finland, a greater proportion of women than men had fair or poor SRH [47]. In contrast to the above observations, Dangi [48] found in Nepal, that females were significantly more likely to rate their health as good compared to males. Given that women have higher life expectancies, the likelihood exists that they endure unhealthy conditions which may potentially influence their functionality, and thus a relatively higher poor SRH [49].

Another potential explanation for the preponderance of negative self-reporting among females may be linked to their relatively lower economic and social status especially in lower-income countries, which restrict or limit their access to health as compared to men [50] [51]. This reinforces the need for gender-based interventions aimed at improving health and reducing gender inequalities in such settings.

Increasing age, predicted an increased odd for poor SRH, those 80 years and over, were 3.85 times likely to have reported poor SRH. This supports the notion that “as the individual’s age increases the likelihood for reporting poor SRH also increases” [45]. This observation is corroborated by other studies, which found that the prevalence of poor or very poor SRH increased with advancing age, and was worse among the very older age groups (75 years and above) [52].

Interestingly also, the geographical location of the older adults influenced their level of SRH in that, rural-dwelling older adults had a higher prevalence of poor SRH. This could potentially be because older adults in urban areas have better average living conditions, increased exposure to health information, better perceptions of health and quality of life compared to those in rural settings; which aligns with findings reported in Malaysia, where residence (rural vs. urban) was shown to have a significant influence on SRH [53]. In addition, the rural-urban disparity observed in this analysis is in consonance with that of the National Health and Nutrition Examination Survey among older adults (65 years and older) in the United States, where, rural-dwelling older adults had lower social functioning than their urban counterparts [54].

In our analysis, adults who were not working experienced poorer SRH (54.9%) and had greater odds of having poor SRH. This is in consonance with the WHO finding that persons in employment are healthier (more especially those who have more control over their working conditions) [55]. Similarly, Asfar et al [56] in their study among Syrian population observed that unemployed participants reported poorer SRH.

Intuitively, underweight (due to malnutrition or ill-health) among older adults is a risk factor for diverse negative health outcomes, including mortality [57]. This was supported by the current analysis which demonstrated a high prevalence of SRH among older adults who were underweight (45.9%), i.e. underweight was found to be a significant predictor of poor SRH. This observation is in line with findings from previous studies indicating that lifestyle factors including being underweight is associated with poor SRH [53] and severe obesity is associated with increased disability and poorer health status. [58]

Another interesting observation from this analysis is the influence of marriage on poor SRH. Older adults who were separated or widowed were found to be more likely to have poor SRH. Marriage is largely considered to be beneficial for health, and that individuals divorced or having never married tend to have poorer health status [59]. This has been observed in Africa, (that single, widowed, separated or divorced older persons displayed significantly poor SRH compared to the married [45] as well as in Europe (that widows/widowers reported worse SRH [60]. Marriage may offer a partner (especially women) a buffer for poor health possibly through greater access to social support and other resources that marriage offers [61].

### Functional difficulty

The effect of SRH on FD has been established in many previous studies. Some looked at SRH effect on psychological FD [62], others look at executive functioning involving neuropsychological tests FD [63] whiles Lollar and colleagues, assessed physical functional difficulties and health conditions among children [64]. In all, SRH assessment on FD is crucial and may strengthen decisions about physical and psychological treatment planning, health and social policy [64].

Aside from these factors, higher levels of physical activity have been shown to be a significant predictor of SRH [65]. Some physically inactive older adults in Ghana reported difficulties in standing for a long period of time, performing households responsibilities, participating in communities’ activities, carrying any load, walking for a long distance, and eating. This is particularly important for the rural-dwelling older adults in Ghana, where their livelihoods involve walking quite long distances to the farm, performing physically tasking activities and carrying some farm produce home for food and self-sustenance almost on daily basis. The fate and challenges of the older adult with FD in such a setting is imperative.

Functioning among older adults usually decline over time [66] and functional disability/difficulty is common [67]. Generally, over two-thirds of all the older adults were found in this analysis to be suffering from some level of functioning difficulties. This is potentially attributable to the notion that at old age quality of life and well-being reduce [68] and the older adults’ ability to function in daily life become sub-optimal. Moreover, at the individual level, functional status declines and co-morbid conditions and disabilities also reduce the older persons’ independence and ability to enjoy an active social life [69] [46]. Thus, functional difficulty may gravely affect their activities of daily living, health, nutrition, and social functioning.

## Limitations

This analysis used only self-reporting by the older adults, a combination of other methods of ascertainment could probably have yielded additional information. In addition, the nature of the data and the modeling approach did not allow for assigning causation effects.

## Conclusion

Poor SRH is more pronounced among older adult females in Ghana. Some determinants of poor SRH include; age, geographical location (urban vs. rural), marital status, religion, and employment status. This provides pointers to important sociodemographic determinants with implications on the social function of older adults and in line with the theme of the national aging policy of 2010, ‘ageing with security and dignity’ and ultimately in the national quest to achieve the Sustainable Development Goals by 2030.

## Abbreviations

AOR: Adjusted Odd Ratio
CEM: Coarsened Exact Matching
CI: Confidence Interval
FD: Functional difficulty
MoH: Ministry of Health
PR: Prevalence Ratio
SAGE: Study on Global Ageing and Adult Health
SRH: Self-rated Healthy
WHO: World Health Organization

## References

[1] Fonta CL, Nonvignon J, Aikins M, Nwosu E, Aryeetey GC (2017) Predictors of self-reported health among the elderly in Ghana: a cross sectional study. BMC Geriatr 17:171 10.1186/s12877-017-0560-y 28760156 PMC5537992

[2] Nery Guimarães JM, Chor D, Werneck GL, Carvalho MS, Coeli CM, Lopes CS, Faerstein E (2012) Association between self-rated health and mortality: 10 years follow-up to the Pró-Saúdecohort study. BMC Public Health 12:676 10.1186/1471-2458-12-676 22905737 PMC3491020

[3] Mora PA, DiBonaventura MD, Idler E, Leventhal EA, Leventhal H (2008) Psychological Factors Influencing Self-Assessments of Health: Toward an Understanding of the Mechanisms Underlying How People Rate Their Own Health. Ann Behav Med 36:292–303 10.1007/s12160-008-9065-4 18937021

[4] Onadja Y, Bignami S, Rossier C, Zunzunegui M-V (2013) The components of self-rated health among adults in Ouagadougou, Burkina Faso. Popul Health Metr 11:15 10.1186/1478-7954-11-15 23926951 PMC3750468

[5] Ocampo JM (2010) Self-rated health: Importance of use in elderly adults. Colomb Médica 41:275–289

[6] WHO (2010) Frequently asked questions: What is the WHO definition of health? In: WHO. http://www.who.int/suggestions/faq/en/. Accessed 15 Jan 2019

[7] Mavaddat N, Kinmonth AL, Sanderson S, Surtees P, Bingham S, Khaw KT (2011) What determines Self-Rated Health (SRH)? A cross-sectional study of SF-36 health domains in the EPIC-Norfolk cohort. J Epidemiol Community Health 65:800–806 10.1136/jech.2009.090845 20551149

[8] Schnittker J (2005) When Mental Health Becomes Health: Age and the Shifting Meaning of Self-Evaluations of General Health. Milbank Q 83:397–423 10.1111/j.1468-0009.2005.00407.x 16201998 PMC2690150

[9] Smith PM, Glazier RH, Sibley LM (2010) The predictors of self-rated health and the relationship between self-rated health and health service needs are similar across socioeconomic groups in Canada. J Clin Epidemiol 63:412–421 10.1016/j.jclinepi.2009.08.015 19926448

[10] Akyol Y, Durmuş D, Doğan C, Bek Y, Cantürk F (2010) Quality of life and level of depressive symptoms in the geriatric population. Arch Rheumatol 25:165–173

[11] WHO (2017) Health situation and trend assessment: Elderly population. In: SEARO. http://www.searo.who.int/health_situation_trends/data/chi/elderly-population/en/. Accessed 17 Aug 2018

[12] Biritwum R, Mensah G, Yawson AE, Minicuci N (2013) Study on global AGEing and adult health (SAGE) Wave 1: The Ghana National Report. 113

[13] St-Onge M-P (2005) Relationship between body composition changes and changes in physical function and metabolic risk factors in aging. Curr Opin Clin Nutr Metab Care 8:523–528 16079623

[14] Cesari M, Onder G, Zamboni V, Manini T, Shorr RI, Russo A, Bernabei R, Pahor M, Landi F (2008) Physical function and self-rated health status as predictors of mortality: results from longitudinal analysis in the ilSIRENTE study. BMC Geriatr 8:34 10.1186/1471-2318-8-34 19102751 PMC2628909

[15] Hoeymans N, Feskens EJM, Kromhout D, Van Den Bos GAM (1997) Ageing and the relationship between functional status and self-rated health in elderly men. Soc Sci Med 45:1527–1536 10.1016/s0277-9536(97)00089-0 9351142

[16] Zarini GG, Vaccaro JA, Canossa Terris MA, Exebio JC, Tokayer L, Antwi J, Ajabshir S, Cheema A, Huffman FG (2014) Lifestyle Behaviors and Self-Rated Health: The Living for Health Program. J Environ Public Health. 10.1155/2014/315042 25530764 PMC4228703

[17] Debpuur C, Welaga P, Wak G, Hodgson A (2010) Self-reported health and functional limitations among older people in the Kassena-Nankana District, Ghana. Glob Health Action. 10.3402/gha.v3i0.2151 20963186 PMC2957305

[18] Duboz P, Boëtsch G, Gueye L, Macia E (2017) Self-rated health in Senegal: A comparison between urban and rural areas. PLoS ONE. 10.1371/journal.pone.0184416 28886107 PMC5590920

[19] Coumé M, Touré K, Faye A, Fall S, Pouye A, Diop TM (2014) Facteurs prédictifs de mauvaise santé perçue chez les femmes âgées sénégalaises. Cah Année Gérontologique 6:90–95

[20] Macia E, Duboz P, Montepare JM, Gueye L (2012) Age identity, self-rated health, and life satisfaction among older adults in Dakar, Senegal. Eur J Ageing 9:243–253 10.1007/s10433-012-0227-7 28804424 PMC5547411

[21] Chola L, Alaba O (2013) Association of Neighbourhood and Individual Social Capital, Neighbourhood Economic Deprivation and Self-Rated Health in South Africa–a Multi-Level Analysis. PLOS ONE 8:e71085 10.1371/journal.pone.0071085 23976923 PMC3743525

[22] Lau YK, Ataguba JE (2015) Investigating the relationship between self-rated health and social capital in South Africa: a multilevel panel data analysis. BMC Public Health. 10.1186/s12889-015-1601-0 25884690 PMC4373512

[23] Ogunyemi AO, Olatona FA, Odeyemi KA (2018) Assessment of factors affecting self-rated health among elderly people in Southwest Nigeria. Niger Postgrad Med J 25:73–78 10.4103/npmj.npmj_14_18 30027917

[24] Ogwumike OO, Adeniyi AF, Orogbemi OO (2016) Physical activity level of postmenopausal women in Nigeria: Association with self-rated health status, overall obesity, and abdominal obesity. Women Health 56:487–501 10.1080/03630242.2015.1101735 26479971

[25] Olawa BD, Adebayo SO, Mokuolu BO, Umeh CS, Omolayo BO (2018) Physical health burdens and emotional distress in later life: the mediating effects of self-rated health. Aging Ment Health 1–7 10.1080/13607863.2018.1506748 30449143

[26] Onadja Y, Bignami S, Rossier C, Zunzunegui M-V (2013) The components of self-rated health among adults in Ouagadougou, Burkina Faso. Popul Health Metr 11:15 10.1186/1478-7954-11-15 23926951 PMC3750468

[27] Blomstedt Y, Souares A, Niamba L, Sie A, Weinehall L, Sauerborn R (2012) Measuring self-reported health in low-income countries: piloting three instruments in semi-rural Burkina Faso. Glob Health Action. 10.3402/gha.v5i0.8488 22833712 PMC3404415

[28] Debpuur C, Welaga P, Wak G, Hodgson A (2010) Self-reported health and functional limitations among older people in the Kassena-Nankana District, Ghana. Glob Health Action 3:2151 10.3402/gha.v3i0.2151 PMC2957305 20963186

[29] Gyasi RM, Phillips DR (2018) Gender, self-rated health and functional decline among community-dwelling older adults. Arch Gerontol Geriatr 77:174–183 10.1016/j.archger.2018.05.010 29787956

[30] Saevareid HI, Thygesen E, Nygaard HA, Lindstrom TC (2007) Does sense of coherence affect the relationship between self-rated health and health status in a sample of community-dwelling frail elderly people? Aging Ment Health 11:658–667 10.1080/13607860701368513 18074253

[31] Fonta CL, Nonvignon J, Aikins M, Nwosu E, Aryeetey GC (2017) Predictors of self-reported health among the elderly in Ghana: a cross sectional study. BMC Geriatr 17:171 10.1186/s12877-017-0560-y 28760156 PMC5537992

[32] Nielsen TH (2016) The Relationship Between Self-Rated Health and Hospital Records. Health Econ 25:497–512 10.1002/hec.3167 25702929

[33] Ibraheem AB, Ibraheem WA, Adebusoye L (2014) The relationship between self-reported health status and spirituality among adult patients attending general outpatient clinic of tertiary hospital in Ibadan. Ann Ib Postgrad Med 12:31–37 25332698 PMC4201931

[34] WHO (2013) Health statistics and information systems: SAGE wave 1. In: WHO. http://www.who.int/healthinfo/sage/cohorts/en/. Accessed 12 Nov 2018

[35] Biritwum RB, Yawson AE, Mensah G, Minicuci N (2015) Ghana: Study on global AGEing and adult health (SAGE) Wave 1 National Report. ResearchGate. 10.13140/RG.2.1.4653.4564

[36] Kowal P, Chatterji S, Naidoo N, et al (2012) Data Resource Profile: The World Health Organization Study on global AGEing and adult health (SAGE). Int J Epidemiol 41:1639–1649 10.1093/ije/dys210 23283715 PMC3535754

[37] Lim W-Y, Ma S, Heng D, Bhalla V, Chew SK (2007) Gender, ethnicity, health behaviour & self-rated health in Singapore. BMC Public Health 7:184 10.1186/1471-2458-7-184 17655774 PMC1976324

[38] Jann B (2004) ALPHAWGT: Stata module to compute Cronbach’s alpha for weighted data. Boston College Department of Economics

[39] Satefano MI, Gary K, Giuseppe P (2009) CEM: Coarsened Exact Matching Software. https://gking.harvard.edu/cem. Accessed 12 Nov 2018

[40] Iacus SM, King G, Porro G (2012) Causal Inference without Balance Checking: Coarsened Exact Matching. Polit Anal 20:1–24

[41] Guarcello MA, Levine RA, Beemer J, Frazee JP, Laumakis MA, Schellenberg SA (2017) Balancing Student Success: Assessing Supplemental Instruction through Coarsened Exact Matching. Technol Knowl Learn 22:335–352

[42] Muennig P, Masters R, Vail D, Hakes J (2017) The effects of New York City’s coordinated public health programmes on mortality through 2011. Int J Epidemiol 46:1239–1248 10.1093/ije/dyw290 28031310 PMC6251557

[43] Chola L, Alaba O (2013) Association of Neighbourhood and Individual Social Capital, Neighbourhood Economic Deprivation and Self-Rated Health in South Africa–a Multi-Level Analysis. PLOS ONE 8:e71085 10.1371/journal.pone.0071085 23976923 PMC3743525

[44] Borim FSA, Neri AL, Francisco PMSB, de Azevedo Barros MB (2014) Dimensions of self-rated health in older adults. Rev Saude Publica 48:714–722 10.1590/S0034-8910.2014048005243 25372161 PMC4211567

[45] Cau BM, Falcão J, Arnaldo C (2016) Determinants of poor self-rated health among adults in urban Mozambique. BMC Public Health 16:20–24 10.1186/s12889-015-2680-7 27553080 PMC4995828

[46] Fuentes-Garcia A, Moreno X, Albala C, Lera L, Sa H (2017) The role of gender in the association between self-rated health and mortality among older adults in Santiago, Chile: A cohort study. PLoS ONE 12:1–10 10.1371/journal.pone.0181317 PMC5515418 28719627

[47] Bardage C, Pluijm SMF, Pedersen NL, Deeg DJH, Jylhä M, Noale M, Blumstein T, Otero Á (2005) Self-rated health among older adults: A cross-national comparison. Eur J Ageing 2:149–158 10.1007/s10433-005-0032-7 28794727 PMC5547684

[48] Dangi A (2016) Factors Associated with Self-Rated Health Among Elderly People Living in Old Age Homes of Kathmandu Valley, Nepal. Oslo and Akershus University College of Applied Sciences

[49] Séculi E, Fusté J, Brugulat P, Juncà S, Rué M, Guillén M (2001) Percepción del estado de salud en varones y mujeres en las últimas etapas de la vida. Gac Sanit SESPAS 15:217–223 10.1016/s0213-9111(01)71550-6 11423025

[50] Chola L, Alaba O (2013) Association of Neighbourhood and Individual Social Capital, Neighbourhood Economic Deprivation and Self- Rated Health in South Africa–a Multi-Level Analysis. 10.1371/journal.pone.0071085 23976923 PMC3743525

[51] Ocampo osé M (2010) Self-rated health: Importance of use in elderly adults. Colomb MÉDICA 41:1–9

[52] Golini N, Egidi V (2016) The Latent Dimensions of Poor Self-Rated Health: How Chronic Diseases, Functional and Emotional Dimensions Interact Influencing Self-Rated Health in Italian Elderly. Soc Indic Res 128:321–339

[53] Chan YY, Teh CH, Lim KK, Lim KH, Yeo PS, Kee CC, Omar MA, Ahmad NA (2015) Lifestyle, chronic diseases and self-rated health among Malaysian adults: results from the 2011 National Health and Morbidity Survey (NHMS). BMC Public Health 1–12 10.1186/1471-2458-15-1 26246019 PMC4527234

[54] Baernholdt M, Yan G, Hinton I, Rose K, Mattos M (2012) Quality of Life in Rural and Urban Adults 65 Years and Older: Findings From the National Health and Nutrition Examination Survey. J Rural Health 28:339–347 10.1111/j.1748-0361.2011.00403.x 23083080 PMC3615459

[55] WHO Health impact assessment: The determinants of health. In: WHO. https://www.who.int/hia/evidence/doh/en/. Accessed 21 Jan 2019

[56] Asfar T, Ahmad B, Rastam S, Mulloli TP, Ward KD, Maziak W (2007) Self-rated health and its determinants among adults in Syria: A model from the Middle East. BMC Public Health 7:1–9 10.1186/1471-2458-7-1 17651491 PMC1976325

[57] Naseer M, Forssell H, Fagerström C (2016) Malnutrition, functional ability and mortality among older people aged⩾ 60 years: a 7-year longitudinal study. Eur J Clin Nutr 70:399 10.1038/ejcn.2015.196 26603879

[58] Imai K, Gregg EW, Chen YJ, Zhang P, Rekeneire N De, Williamson DF (2008) The Association of BMI With Functional Status and Self-rated Health in US Adults. Obes J 16:402–408 10.1038/oby.2007.70 18239651

[59] Lindström M (2009) Marital status, social capital, material conditions and self-rated health: A population-based study. Health Policy 93:172–179 10.1016/j.healthpol.2009.05.010 19692141

[60] Hardy M, Acciai F, Reyes A (2014) How Health Conditions Translate into Self-Ratings: A comparative study of older adults across Europe. J Health Soc Behav 55:320–341 10.1177/0022146514541446 25138200 PMC4669051

[61] Åhs A, Westerling R (2006) Self-rated health in relation to employment status during periods of high and of low levels of unemployment. Eur J Public Health 16:294–304 10.1093/eurpub/cki165 16260444

[62] Christian LM, Iams J, Porter K, Leblebicioglu B (2013) Self-rated Health among Pregnant Women: Associations with Objective Health Indicators, Psychological Functioning, and Serum Inflammatory Markers. Ann Behav Med Publ Soc Behav Med. 10.1007/s12160-013-9521-7 23765366 PMC3797861

[63] Jung MS, Lee KS, Kim M, Yun H (2019) Gender-Specific Relationship Between Executive Function and Self-Rated Health. Osong Public Health Res Perspect 10:93–101 10.24171/j.phrp.2019.10.2.08 31065536 PMC6481577

[64] Lollar DJ, Hartzell MS, Evans MA (2012) Functional Difficulties and Health Conditions Among Children With Special Health Needs. Pediatrics 129:e714–e722 10.1542/peds.2011-0780 22371461

[65] Arnadottir SA, Gunnarsdottir ED, Stenlund H, Lundin-Olsson L (2011) Determinants of self-rated health in old age: A population-based, cross-sectional study using the International Classification of Functioning. BMC Public Health 11:670 10.1186/1471-2458-11-670 21867517 PMC3175467

[66] Spalter T, Brodsky J, Shnoor Y (2014) Improvements and decline in the physical functioning of israeli older adults. Gerontologist 54:919–929 10.1093/geront/gnt084 23969256

[67] Colón-Emeric CS (2014) Functional Decline in Older Adults—American Family Physician. Am Fam Physician 88:388–394 PMC3955056 24134046

[68] Steptoe A, Deaton A, Stone AA (2015) Psychological wellbeing, health, and ageing. The Lancet 385:640–8 10.1016/S0140-6736(13)61489-0 PMC4339610 25468152

[69] Paterson HdH, Warburton DE (2010) 3D FE Model of the feline patellofemoral joint contact. Int J Behav Nutr Phys Act Ity 7:190

